# The Impact of Skill Level on the Integration of Information and Post-Error Adjustment during Action Anticipation in Basketball

**DOI:** 10.3390/bs14050423

**Published:** 2024-05-20

**Authors:** Zhefu Chen, Danlei Wang, Wenxuan Fang, Mengkai Luan

**Affiliations:** 1 School of Kinesiology and Health Promotion, Dalian University of Technology, Dalian 116024, China; chenzhefu@dlut.edu.cn; 2 School of Psychology, Shanghai University of Sport, Shanghai 200438, China; 2221920007@sus.edu.cn; 3 School of Athletic Performance, Shanghai University of Sport, Shanghai 200438, China; fangwenxuan@sus.edu.cn

**Keywords:** action anticipation, contextual prior information, kinematic information, post-error adjustment, Bayesian

## Abstract

The present study examined the impact of skill level on the integration of contextual prior information and kinematic information alongside post-error adjustment during action anticipation in basketball. Twenty-three collegiate basketball players and twenty-three control participants engaged in anticipating as quickly and accurately as possible the outcomes of free throws, utilizing video clips depicting basketball players’ actions, both with and without contextual prior information. Anticipatory performance and the difference in anticipatory performance following errors and correct responses were analyzed based on skill level and the congruency of contextual prior information (none, congruent, and incongruent). The findings revealed that the congruency of contextual prior information significantly affects action anticipation, with skill level moderating this effect. Moreover, skill level influenced the congruency effect on accuracy discrepancies between post-error and post-correct trials during action anticipation, with controls showing greater sensitivity to previous trial performance compared to experts. These results provide further evidence for the notion that individuals employ Bayesian reliability-based strategies to integrate different information sources and underscore the role of skill level in adjusting anticipatory judgments following errors during action anticipation. These insights contribute to a deeper understanding of the cognitive and behavioral mechanisms that differentiate skill levels in action anticipation, potentially guiding the development of targeted training interventions.

## 1. Introduction

Action anticipation is a critical ability in competitive sports, essential for predicting and preparing for upcoming action outcomes under high-pressure and time-constrained conditions [1]. Action anticipation relies on various sources of information, encompassing kinematic information and contextual prior information [2,3]. Individuals may employ Bayesian reliability-based strategies to integrate and weight the importance of different information sources during action anticipation in order to make the most accurate anticipatory decisions [2]. Distinct skill levels are likely to attach differing degrees of significance to these information sources, consequently shaping the integration process during action anticipation [4,5]. Furthermore, when examining the use of contextual prior information and kinematic information during action anticipation, post-error adjustment is often overlooked. Detecting errors can trigger deliberate post-error adjustments aimed at mitigating such occurrences in the future [6]. Skill level further contributes to the formulation of distinct patterns of post-error adjustment. In the current study, we examined the impact of skill level on the integration of contextual prior information and kinematic information alongside post-error adjustment dynamics within the context of action anticipation.

### 1.1. Integration of Kinematic and Contextual Prior Information in Action Anticipation

Kinematic information, primarily visual, provides vital perceptual cues during action execution, such as the opponent’s posture [7], equipment trajectories [8], or player motions in team sports [9]. A multitude of investigations have affirmed that skilled athletes adeptly extract kinematic information to deduce forthcoming outcomes, outperforming control participants or less experienced athletes [10,11,12,13]. Moreover, it has been found that the action observation network (AON) in the brain plays a significant role in processing kinematic information, forming internal models that aid in action anticipation based on perceptual motor experiences [14,15,16,17,18,19]. Contextual prior information encompasses knowledge about event probabilities in specific game situations, including scores [20], opponent positions [21], and movement tendencies [22]. Previous research highlights the interactive nature of kinematic and contextual prior information in influencing action anticipation performance [4,23,24]. Studies have shown that skilled athletes adapt their reliance on contextual prior versus kinematic information during action anticipation based on the timing of occlusion in visual stimuli, demonstrating a nuanced integration process [4].

In recent years, probabilistic inference Bayesian models have emerged as a promising framework for comprehending how contextual prior and kinematic information is integrated during action anticipation [2,5]. According to Bayesian theory, these information sources are melded in a probabilistic manner to reduce uncertainty in anticipatory judgments. This suggests that the impact of contextual prior information on action anticipation is influenced by the reliability of the accompanying kinematic information, and vice versa [25]. In basketball, athletes must combine contextual prior information, such as a player’s shooting history and previous shot locations, with real-time kinematic information to enhance the accuracy of their anticipatory judgments. While studies on kinematic information in basketball exist [10,16,26,27], exploring the impact of contextual prior information on anticipatory performance across different skill levels remains an uncharted domain.

### 1.2. Post-Error Adjustment in Action Anticipation

Error monitoring refers to the ability to detect and evaluate discrepancies between expected and actual outcomes or performances [28]. It involves neural processes that identify errors and prompt subsequent adjustments in cognitive control [6]. Recent studies have demonstrated that specific motor experiences within a particular domain play a crucial role in detecting errors during action anticipation [12,29,30]. Notably, the detection of errors could trigger adjustments in order to avoid these errors in subsequent trials (post-error adjusting) [31,32]. Despite the extensive exploration of action anticipation, a gap exists in comprehending post-error adjustment within this context, especially its impact on the integration of contextual prior information and kinematic information. When errors occur, individuals may adjust their prediction strategies to improve accuracy by varying the weights assigned to different sources of information. Moreover, there are skill level differences in the ability to extract kinematic information, efficiency of integration, and anticipation strategies [4,10,22]. Skill-specific variations in post-error adjustment concerning the integration of distinct information sources may diverge across skill levels. Addressing these gaps is essential for enhancing our understanding of the cognitive processes underlying action anticipation.

### 1.3. Aims and Hypotheses of This Study

This study examines how skill level influences the integration of contextual prior information and kinematic information as well as post-error adjustments during action anticipation in basketball. Expert basketball players and control participants were recruited to anticipate free throw outcomes using video clips depicting basketball actions with and without contextual prior information. The study considered the congruence between contextual prior information and free throw outcomes, exploring three conditions: none, congruent, and incongruent [22,33]. Anticipatory performance was recorded across these conditions. Skilled basketball players, benefitting from years of deliberate practice, are adept at extracting and prioritizing kinematic information during action anticipation, while control participants lean towards contextual prior information due to lack of motor experience. Therefore, we hypothesize that skill level will significantly influence the integration of contextual prior information and kinematic information during action anticipation in basketball.

Furthermore, the study evaluates post-error adjustments by comparing anticipatory performance after errors and correct responses across different congruency conditions. Expert basketball players are expected to exhibit stable and efficient anticipatory strategies, potentially requiring minimal adjustments through error monitoring. In contrast, control participants’ integration strategies may be inconsistent. The detection of errors could prompt adaptations in anticipatory strategies, potentially leading controls to diminish the emphasis on contextual prior information during post-error action anticipation compared to post-correct scenarios. Thus, we hypothesize that the impact of congruency on post-error adjustments will be moderated by skill level.

## 2. Materials and Methods

### 2.1. Participants

A total of 23 collegiate players and 23 control participants were recruited in this experiment for a monetary reward (see demographic information of participants in Table 1). With the sample size used in the study, effect sizes (f) greater than 0.19 can be detected with a power level of 0.80, an alpha level of 0.05, and correlations between repeated measures of 0.5 in a sensitivity analysis assuming a 2 × 3 repeated measures ANOVA design, as computed using G*Power [34]. The collegiate players were from the Chinese University Basketball Association (CUBA), a highly competitive basketball league for Chinese university students. They practiced for more than or equal to 5 days a week and more than or equal to 4 h each practice session during the last 6 years. They are qualified as a National Player at the second grade or above. According to the classification by Macnamara et al. [35], participants competing at the national level or higher are considered elite. Therefore, our athlete participants have ample grounds to be deemed experts. The control participants were recruited from the general university student population with no prior basketball experience and minimal exposure to the sport in their daily lives. All participants had normal or corrected-to-normal vision and had no knowledge of the expected outcome of this experiment. None of the participants had a history of alcohol or drug dependence or any neurological disorders. One player was left-handed. Informed written consent in accordance with the Declaration of Helsinki was obtained from all participants prior to the experiment. The study was approved by the local ethics committee of Shanghai University of Sport (No. 102772022RT069).

### 2.2. Stimuli

**Figure 1:**
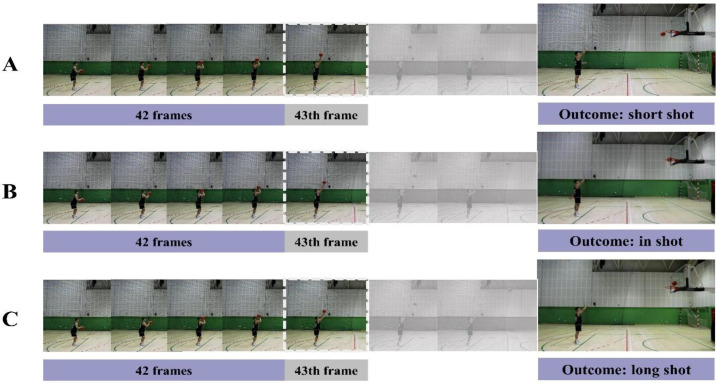
Video clips. (**A**) The player altered the kinematics so that the ball’s trajectory did not reach the position of the basket (short shot); (**B**) the player performed a prototypical shooting motion so that the ball entered the basket without touching the basket (in shot); (**C**) the player altered the kinematics so that the ball’s trajectory went beyond the position of the basket (long shot). Please note that each occlusion video clip stimulus began 42 frames before ball release and ended 2 frames after ball release.

**Figure 2:**
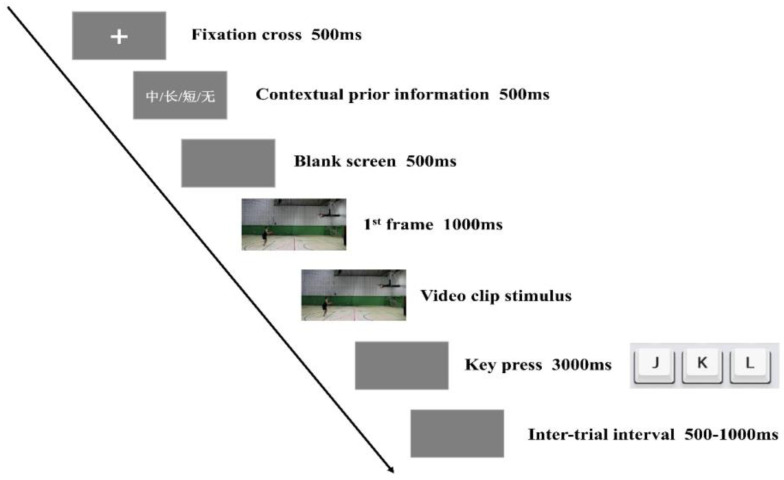
The procedure of each trial. The Chinese characters “短”, “中”, “长” or “无” respectively represent the meanings of “short”, “in”, “long”, and “none” in English.

**Figure 3:**
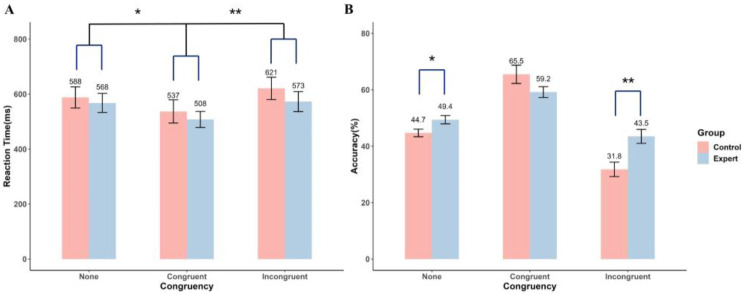
Reaction time (**A**) and accuracy (**B**) in action anticipation as a function of group and congruency condition. * *p* < 0.05, ** *p* < 0.01.

The stimuli were self-recorded and self-produced occlusion video clips of basketball players’ free throw actions. We recorded video clips of two right-handed professional basketball players (one male and one female) performing free throws using a digital video camera (60 Hz; Canon EOS R6; positioned 6 m from a sagittal viewpoint). We asked each player to complete three distinct types of free throw: (1) altering the kinematics so that the ball’s trajectory would fall short of reaching the position of the basket (short shot; Figure 1A); (2) performing a prototypical shooting motion ensuring the ball enters the basket without touching the basket (in shot; Figure 1B); (3) altering the kinematics to propel the ball’s trajectory to go beyond the position of the basket (long shot; Figure 1C). Each player continued to make free throws until successfully accomplishing 20 free throws for each of the three types. Subsequently, we edited the video clips using Adobe Premiere Pro CS6 software (Adobe Systems Incorporated, San Jose, CA, USA) to create occlusion video clips. Each occlusion video clip began 42 frames before ball release and ended 2 frames after ball release [26].

### 2.3. Task and Procedure

Participants sat in a dimly lit and soundproof room in front of a 17-inch monitor at an approximate distance of 60 cm. The spatial resolution of the monitor was set at 1024 × 768. Throughout the experiment, participants rested their right index, middle, and ring fingers on the “J”, “K”, and “L” keys, respectively. The experimental tasks were programmed and implemented using E-prime software 3.0 (Psychology Software Tools, Inc., Sharpsburg, PA, USA).

There were two conditions in this experiment: one with contextual prior information (prior condition) and one without contextual prior information (control condition). Each trial started with the presentation of a central fixation cross for 500 ms. Then, the contextual prior information-related cue (white, 40-point) was displayed for 500 ms at the center of the monitor, followed by a blank screen lasting 500 ms. In the prior condition, the cue was “短”, “中”, or “长” (Chinese for “short”, “in”, “long”, respectively), indicating a short shot, in shot, or long shot, respectively. The cues may or may not have been in line with the outcome of the upcoming video clip (target stimulus). Participants were informed that the cues might not always accurately provide contextual prior information and that the probability of congruent trials would be greater than incongruent trials. Previous research using similar stimuli and tasks found that the outcome prediction accuracy of basketball players in a condition without contextual prior information was 46.7% [26]. In order to make participants believe and use the contextual prior information but prevent them from using only the contextual prior information and ignoring the kinematic information, we set the proportion of trials with consistent cues and outcomes to 53.3% (8/15) of all trials, which was slightly higher than 46.7%. In the control condition, the cue was “无” (Chinese for “none”), indicating the absence of contextual prior information. Subsequently, the first frame of the video was exhibited for 1000 ms, followed by the presentation of the video clip stimulus. After the video clip stimulus ended, participants were requested to anticipate the outcome of the free throw and press the corresponding key as quickly and accurately as possible: “J” for short shot, “K” for in shot, and “L” for long shot [10,12,13,26]. After the key press or 3000 ms after the video clip stimulus finished (in case of no key press), participants received feedback about the correctness of their response for 1000 ms (correct, error, or too slow). The interval between consecutive trials varied between 500 and 1000 ms (See Figure 2).

Participants first performed twelve practice trials to familiarize themselves with the experimental task, four trials for each of the four distinct cues. After the familiarization trials, participants performed four blocks of 60 trials, two for each condition. The instructions for each block were displayed as text on the screen at the beginning of each block. There was a brief break between blocks. The order of conditions followed an ABBA pattern to minimize the influence of potential learning effects. The same set of 60 video clip stimuli was randomly presented across all blocks.

### 2.4. Data Analysis

Reaction time (RT) was defined as the time between the end of the video presentation and the key press. Only RTs associated with correct responses were further analyzed. For each participant, RTs below 100 ms or greater than 3 standard deviations above the condition mean were considered outliers and thus excluded from further analyses (0.3%). Trials with consistent cues and outcomes of the video clips (i.e., congruent trials) and trials with inconsistent cues and outcomes of the video clips (i.e., incongruent trials) were analyzed separately. The mean RT and accuracy per participant and congruency condition were calculated. The mean RTs and accuracies were analyzed with 2 (Group: Expert and Control) × 3 (Congruency: none, congruent, and incongruent) mixed ANOVAs with Group as the between-subject factor and Congruency as the within-subject factor.

To explore the post-error adjustment, mean RTs and accuracies were calculated based on the congruency in trial n and the correctness in trial n − 1 for each participant. The difference between mean RTs after errors and those after correct responses and the difference between accuracies following error and those following correct responses were then computed across various congruency conditions. Differences in mean RT and accuracy between post-error trials and post-correct trials were analyzed with 2 (Group: Expert and Control) × 3 (Congruency: none, congruent, and incongruent) mixed ANOVAs with Group as the between-subject factor and Congruency as the within-subject factor.

For all analyses, effect sizes are reported as partial eta-squared ($\eta_{p}^{2}$) values. In cases where the sphericity assumption was violated according to Mauchly’s test, the Greenhouse–Geisser correction was employed to adjust degrees of freedom. Furthermore, in the presence of the main effect of congruency condition or any interaction effect, Bonferroni-adjusted post-hoc multiple comparisons were conducted.

## 3. Results

### 3.1. Reaction Time

Figure 3A displays the results of the ANOVA on mean RT, indicating a significant main effect of Congruency (F(2.00, 87.90) = 7.81, *p* = 0.001, $\eta_{p}^{2}$ = 0.15). Bonferroni-adjusted post-hoc multiple comparisons showed that the RTs in the congruent condition were significantly faster than in the other two conditions (*p* < 0.018). The main effect of Group (F(1, 44) = 0.46, *p* = 0.50) and the Group × Congruency interaction (F(2.00, 87.90) = 0.28, *p* = 0.76) did not approach significance.

### 3.2. Accuracy

Figure 3B illustrates the results of the ANOVA on accuracy, demonstrating a significant main effect of Congruency (F(1.15, 54.57) = 57.12, *p* < 0.001, $\eta_{p}^{2}$ = 0.56) and a significant Group × Congruency interaction (F(1.15, 54.57) = 7.56, *p* = 0.005, $\eta_{p}^{2}$ = 0.15). Bonferroni-adjusted post-hoc multiple comparisons showed that there were significant differences in accuracy between groups in the none and incongruent contextual prior information conditions (*p* < 0.023). In contrast, in the congruent condition, the accuracy of experts did not significantly differ from that of controls (*p* = 0.11). The main effect of Group was not statistically significant (F(1, 44) = 3.72, *p* = 0.06).

### 3.3. Analysis of Post-Error Adjustment

**Figure 4:**
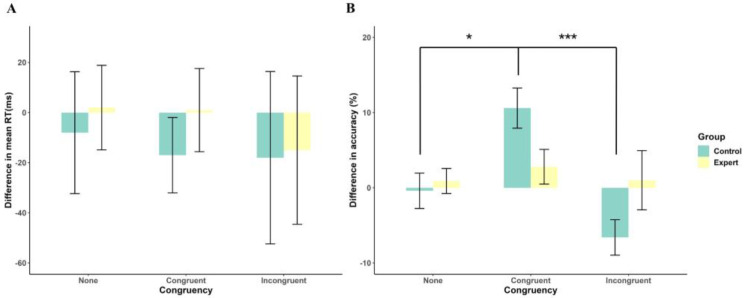
Difference in reaction time (**A**) and accuracy (**B**) between post-error trials and post-correct trials as a function of group and congruency condition. * *p* < 0.05, *** *p* < 0.001.

One control participant submitted all wrong responses in the incongruent trial, which preceded an error in trial n − 1. Thus, we had 22 control participants’ mean RT data for this analysis. Figure 4A displays the results of the ANOVA on the difference in mean RT between post-error trials and post-correct trials, demonstrating no significant main effect or interaction effect (Fs < 0.87, *p* > 0.61).

Figure 4B illustrates the results of the ANOVA on difference in accuracy between post-error trials and post-correct trials, indicating a significant main effect of Congruency (F(1.82, 82.80) = 6.26, *p* = 0.004, $\eta_{p}^{2}$ = 0.12) and a significant Group × Congruency interaction (F(1.82, 82.80) = 4.06, *p* = 0.021, $\eta_{p}^{2}$ = 0.08). Bonferroni-adjusted post-hoc multiple comparisons showed that for control participants, the difference in accuracy between post-error trials and post-correct trials in the congruent condition was higher than that in the other two conditions (*p* < 0.016). In contrast, for experts, no significant differences were observed across the three congruency conditions (*p* > 0.90). The main effect of Group was non-significant (F(1, 44) = 0.06, *p* = 0.82).

**Figure 5:**
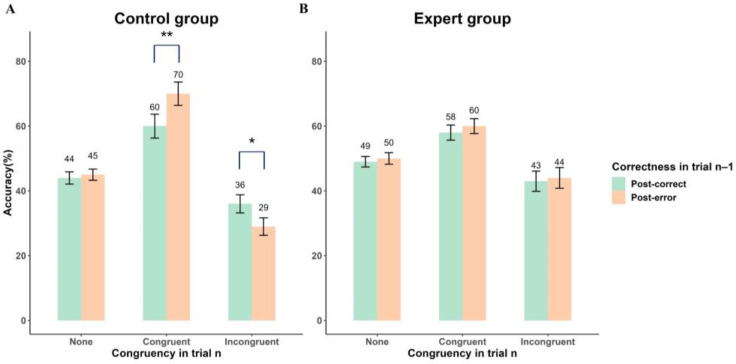
Accuracy in action anticipation as a function of group, congruency condition, and correctness in trial n − 1. (**A**) Data from the control group. (**B**) Data from the expert group. * *p* < 0.05, ** *p* < 0.01.

To investigate differences in the modulation of the congruency as a function of the preceding occurrence of performance errors between different groups, we conducted two ANOVAs on accuracy for the two groups separately, with Correctness in trial n − 1 and Congruency in trial n as factors. For control participants, Figure 5A displays that a significant main effect of Congruency in trial n was observed (F(1.17, 25.69) = 36.09, *p* < 0.001, $\eta_{p}^{2}$ = 0.62). Additionally, a significant Correctness in trial n − 1 × Congruency in trial n interaction was found (F(2.00, 43.88) = 9.79, *p* < 0.001, $\eta_{p}^{2}$ = 0.31). Bonferroni-adjusted post-hoc multiple comparisons showed that in the congruent condition, the accuracy of post-error trials (M = 69.8%, SD = 3.6%) was significantly higher than that of post-correct trials (M = 59.7%, SD = 3.7%; *p* = 0.003). In the incongruent condition, the accuracy of post-error trials (M = 28.9%, SD = 2.7%) was significantly lower than that of post-correct trials (M = 35.6%, SD = 2.8%; *p* = 0.021), and there was no difference between the accuracy of post-error trials (M = 44.7%, SD = 1.7%) and post-correct trials (M = 44.7%, SD = 1.79%) in the none prior condition (*p* = 0.91). However, for experts, as depicted in Figure 5B, the ANOVA showed that neither the main effect of Congruency in trial n nor the Correctness in trial n − 1 × Congruency in trial n interaction approached significance (Fs < 0.80, *p* > 0.38).

## 4. Discussion

The present study examined the impact of skill level on the integration of contextual prior information and kinematic information during anticipation in basketball. Specifically, expert basketball players and control participants predicted the outcome of free throw actions both with and without contextual prior information-related cues. We captured the accuracy and reaction time of their action anticipation as their anticipatory performance. Furthermore, to investigate the impact of skill level on post-error adjustments during action anticipation, the differences in anticipatory performance following errors and correct responses across distinct congruency conditions were computed and analyzed.

### 4.1. Skill Level Impact on Action Anticipation: Integrating Contextual Prior and Kinematic Information

In line with our predictions, as demonstrated in Figure 5, contextual prior information had a significant impact on action anticipation, and this impact was modulated by skill level. Both experts and controls showed faster and more accurate action anticipation with congruent, compared to without, contextual prior information. Meanwhile, the incongruent contextual prior information decreased their accuracy of action anticipation. Notably, experts demonstrated higher accuracy than controls in the none and incongruent contextual prior information conditions. However, there was no difference in accuracy between groups in the congruent contextual prior information condition. These findings support previous research suggesting that probabilistic inference Bayesian modeling accounts for how individuals integrate contextual prior information with kinematic information during action anticipation [2,5,36]. According to Bayesian theory, individuals integrate contextual prior and kinematic information in a probabilistic manner as a way to reduce uncertainty in their anticipation judgments. This entails adjusting the weighting of each type of information source based on its precision or reliability. When one type of information source is less reliable, action anticipation will be more heavily weighted toward the other more reliable type of information source [24]. This dynamic interaction implies that the impact of contextual prior information on action anticipation is contingent on the reliability of the subsequently presented kinematic information, and vice versa [25]. Experts have extensive domain-specific knowledge and experience. Compared to controls, they extract kinematic information better and give more weight to kinematic information during action anticipation. They may be more adept at recognizing the congruency of contextual prior information and can better adjust their anticipatory judgments based on their expertise, which can make their action anticipation be positively influenced by congruent information and less negatively influenced by incongruent information [3]. On the contrary, individuals with less expertise may rely more heavily on the provided contextual prior information due to limited kinematic information processing skills, leading to a higher tendency to follow the given cues. Therefore, while they may have achieved a comparable performance to experts in congruent conditions, their performances were much worse in the incongruent condition. In summary, experts are better equipped to adapt and adjust their anticipatory judgments based on their domain-specific knowledge and superior kinematic information processing skills.

It is worth noting that our results are inconsistent with previous research that has shown experts are more accurate than novices in action anticipation in congruent contextual prior information conditions [4,22]. We consider that these discrepancies could be attributed to the distinct nature of the contextual prior information used. In those studies, the contextual prior information encompasses implicit or tacit knowledge individuals have to acquire through their experiences, learning, and past interactions with similar situations or tasks, such as game situations and field settings [4]. Controls, with limited experience, lack a well-developed mental representation of relevant cues, which may hinder their ability to connect prior information to the current context effectively [22]. In that case, the reliability of both kinematic and contextual prior information may be compromised, resulting in a challenge to achieve accurate anticipation [3]. In contrast, our study employed directly provided prior information, as shown in Figure 2. This information was readily accessible to controls without necessitating prior experience or expertise. This direct provision of information levels the playing field for controls, allowing them to access relevant cues that aid in action anticipation. A study by Wang et al. [33] could provide evidence for our statement. They asked soccer goalkeepers and controls to perform a cue anticipation task to anticipate penalty kick outcomes using directional arrows as contextual prior cues for ball trajectories. These cues could be accurate or inaccurate. They found that goalkeepers showed greater accuracy than controls in neutral and incongruent conditions, while in the congruent condition there was no significant difference in accuracy between groups, which is line with our results [33]. Future research might delve further into understanding how individuals use different types of prior information during action anticipation. This could provide deeper insights into the intricate interplay between skill level, contextual prior information, and kinematic information in action anticipation. Additionally, it is worth considering that the use of a key-press task instead of real-life scenarios might also contribute to the discrepancies between our findings and those of previous research. While our study employed a computer key-press system to assess action anticipation, previous studies have utilized more ecologically valid tasks. For instance, in Gredin et al.’s study [22], the stimulus clips were projected onto a 4.1 × 2.3 m projection screen and participants were positioned four meters from the screen, holding a bespoke response device in each hand to anticipate the direction of the player in possession’s final action. Their experiments may be closer to real-life tasks. This methodological difference could impact the extent to which expertise-related differences in action anticipation are observed. Further investigations could explore how the choice of task modality influences the outcomes of action anticipation studies, providing valuable insights into the factors contributing to the observed discrepancies between our results and those of prior research.

### 4.2. Skill Level Impact on Post-Error Adjustment in Action Anticipation

An important objective of this study was to examine the impact of skill level on post-error adjustment during action anticipation. As we predicted, the effect of congruency on the difference in accuracy between post-error and post-correct trials was modulated by skill level. This effect was manifested in the fact that the controls were more accurate in post-error than post-correct trials in the congruent contextual prior information condition, while showing the opposite results in the incongruent condition, as shown in Figure 5A. On the contrary, the experts’ accuracy was not affected by the preceding trial’s performance, as depicted in Figure 5B. This intriguing finding can potentially be elucidated by two explanations, which are not mutually exclusive. Firstly, a previous study found that the integration of kinematic information and contextual prior information during action anticipation requires certain cognitive demands [37]. Errors can indeed increase the cognitive load for individuals. When individuals encounter errors, they need to engage in additional cognitive processes to detect and analyze the errors [38]. Controls typically require more cognitive resources to process both kinematic and prior information and integrate them for action anticipation than experts do. When controls experience errors in a previous action anticipation, the heightened cognitive load could lead to them not having enough attentional resources available to process all information sources. They may then overemphasize the importance of contextual prior information during subsequent action anticipation, inadvertently marginalizing the role of actual kinematic information, as illustrated in Figure 5A. Secondly, experts usually demonstrate greater confidence in their action anticipation. They have higher self-assurance in their judgments and rely more autonomously on their internal model [16,17,18,19]. When faced with errors, experts are more likely to maintain confidence in their integration strategies, rendering them less susceptible to the influence of errors from the previous trial, as shown in Figure 5B. In contrast, controls may lack the same level of confidence and instead exhibit more cautious and erroneous adjustments in the subsequent action anticipation, aiming to minimize the risk of errors.

These findings offer valuable insights into the cognitive processes underlying the action anticipation abilities of experts and controls, thereby offering potential pathways for tailored training interventions. For controls or novices, cultivating adaptive error processing, reducing cognitive load, and enhancing confidence can improve their action anticipation performance. For experts, the focus may be on maintaining stable integration strategies and self-confidence to enhance the accuracy and efficiency of action anticipation. However, we did not directly measure participants’ cognitive load and confidence during the action anticipation task. It is essential to acknowledge that these potential explanations remain speculative. Future research should consider incorporating additional measures, such as electroencephalography or self-report questionnaires, to gain a more comprehensive understanding of participants’ cognitive load and confidence during action anticipation, which may further elucidate the mechanisms underlying the integration of kinematic and contextual prior information at different skill levels [37,39,40].

Another potential limitation of this study is that our experimental design required participants to press the corresponding key after the completion of the video clip stimulus. In the rules of basketball, players can only jump to compete for the rebound after the free throw is released. Therefore, we used this experimental design which closely resembled real-life sports scenarios to ensure a high ecological validity. However, participants may have already finished their action anticipation during the playback of the action video clips. The absence of differences in reaction times between controls and experts, which is not common in studies of action anticipation [16,41,42], and the lack of post-error slowing in the experiment may indeed be related. Post-error slowing refers to the phenomenon where individuals slow down their reaction times following an error, indicating increased cognitive control and error awareness [32,43,44]. An interesting question to be addressed by future research would be comparing our results with an alternative design of allowing participants to press keys during video clip playback to gain deeper insights into the influence of experimental design on the obtained outcomes. Moreover, it is important to acknowledge the influence of individual characteristics, such as handedness, on the study outcomes. Given that we had only one left-handed participant in our study, the impact of handedness on our experimental results is likely minimal. However, considering the handedness of participants is an intriguing avenue for future research. Handedness has been shown to influence various cognitive and motor functions, and its potential impact on action anticipation abilities warrants further investigation [45]. Future studies could explore how handedness affects the integration of kinematic and contextual prior information during action anticipation tasks, potentially shedding light on individual differences in performance.

## 5. Conclusions

In summary, the present study provides further evidence for the notion that individuals employ Bayesian reliability-based strategies to integrate and weight different information sources during action anticipation [2,46]. Experts’ domain-specific knowledge and superior kinematic information processing skills enable them to better adjust anticipatory judgments based on congruency, while controls rely more heavily on contextual prior information, resulting in reduced performance in scenarios involving incongruent contextual prior information. Furthermore, our findings underscore that experts exhibit stable and effective integration strategies, even after encountering errors in previous trials. In contrast, controls appear to exhibit less efficient processing of different information resources and a lack of confidence, rendering them more susceptible to adjustments following errors. Importantly, the cognitive processes elucidated in our study not only hold potential relevance within the specific domain of sports or the tasks examined but also extend to a diverse array of related activities. The mechanisms by which individuals integrate kinematic and contextual prior information during action anticipation bear relevance to various sports as well as broader domains such as driving, aviation, and other everyday activities necessitating rapid decision-making based on visual cues. Future research could explore these possibilities further and investigate the transferability of our results to other contexts, ultimately contributing to a deeper understanding of the cognitive processes underlying action anticipation.

## Figures and Tables

**Table 1:** Demographic information of participants.

	Experts	Controls
Number	23	23
Age, mean ± SD, years	20.17 ± 1.07	19.8 ± 1.64
Years of training, mean ± SD	8.17 ± 2.17	0
Training frequency (days/week)	5–7	0
Training time (hours/day)	4–5	0
Body mass index, mean ± SD	23.62 ± 2.01	20.38 ± 3.29

## Data Availability

Stimulus materials, raw data, and analysis scripts are available on the Open Science Framework (https://osf.io/rx9vm/).
